# An observational study into the management of arteriomegaly: a call for a revised classification system

**DOI:** 10.1308/003588412X13171221498505

**Published:** 2012-05

**Authors:** JV Barandiaran, TC Hall, I Glaves, N El-Barghouti, EP Perry

**Affiliations:** Scarborough General HospitalUK

**Keywords:** Arteriomegaly, Aneurysm, Vascular ectasia, Progressive disease

## Abstract

**INTRODUCTION:**

Arteriomegaly is the diffuse ectasia of arteries with or without aneurysmal disease. Patients with arteriomegaly have a higher incidence of morbidity including limb loss compared to patients with other arteriopathies. The aim of this observational study was to review the management of these patients in our institution.

**METHODS:**

Radiologists and surgeons prospectively reviewed aortofemoral angiography. Patients with arteriomegaly were identified. Data relating to demographics, mode of presentation, risk factors, type of arteriomegaly, management and progression of disease were analysed.

**RESULTS:**

Arteriomegaly was identified in 1.3% of patients *(n=*69) undergoing lower limb angiography in the study period. Of these, the majority *(n=*67) were men. The mean age was 74 years (range: 60–89 years) and 76% were smokers. Co-morbidities included coronary artery disease (55%), diabetes mellitus (20%), hypertension (45%) and cerebrovascular events (6%). Fortynine patients presented with critical limb ischaemia and eighteen patients were seen electively in the outpatients department with symptoms of intermittent claudication. Data were incomplete for two male patients and were therefore not included. At presentation, 22 patients were classified as Hoi lier type I, 5 were type II and 9 were type III. Thirty-one patients had arteriomegalic vessels but no aneurysmal disease.

After a median follow-up duration of 76 months (range: 6–146 months), 34 patients progressed to type I, 2 to type II and 18 to type III. Thirteen remained without aneurysmal disease. Twenty-nine patients required angioplasty and twenty-eight required bypass surgery during this time. In total, 102 procedures were required for complicated disease. The limb salvage rate was 92%. Although 8 patients in our series died, the remaining 59 are under regular follow up.

**CONCLUSIONS:**

This study illustrates the progressive nature of arteriomegaly. Results of the management of these patients in our institution are similar to those in the literature. We suggest an additional fourth category to Hollier’s classification that describes arteriomegalic disease without aneurysmal degeneration as this, too, deserves special management. Regular follow-up visits and early intervention for patients with arteriomegaly is advocated to reduce the high incidence of morbidity.

Arteriomegaly is a concept that was introduced by René Leriche in 1945.^1^ It is the diffuse ectasia of arteries with or without diffuse aneurysmal disease. It represents a separate clinical entity to multiple isolated arteriosclerotic aneurysms. Multiple isolated arteriosclerotic aneurysms are usually less extensive, have a lower associated mortality and differ in their surgical management compared to arteriomegalics.^2^

Hollier *et al* classified arteriomegaly into three types.^2^ They defined aneurysms as the localised increase in arterial luminal diameter of at least 1.5 times its normal size. Diffuse aneurysmal disease was defined as the presence of aneurysmal disease in three or more separate arterial seg ments described. The aims of this classification were to identify specific risk factors, create a standard terminology and aid its surgical approach:

*Type I·.* Aneurysms present in the aorta, iliac and common femoral arteries, with arteriomegaly of the superficial femoral and popliteal arteries

*Type II·.* Aneurysms in the common femoral, superficial and popliteal arteries, with arteriomegaly of the aorta and iliac arteries

*Type III:* Aneurysms in the aortoiliac, femoral and popliteal arteries, with arteriomegaly of intervening arteries that are not specifically aneurysmal

Arteriomegaly is a progressive disease with different modes of presentation. Although a relatively rare disorder, it is associated with a high incidence of morbidity and mortality. The aim of this observational study was to review the management of this condition in a district hospital.

## Methods

An observational study was designed to analyse the management of arteriomegaly in our institution between 1995 and 2009. A computer database was used to identify all patients who underwent aortofemoral angiography by conventional angiography, computed tomography (CT) angiography or magnetic resonance angiography (MRA).

All patients with an angiographic diagnosis of arteriomegaly made by any radiological modality and reported by a consultant radiologist were included in the study. Records were identified by ‘text searching’ our computer database of radiological reports. All imaging was reviewed by a consultant radiologist to reconfirm the diagnosis of arteriomegaly. The medical records of these patients were reviewed.

Data relating to demographics, risk factors, mode of presentation, investigations, arteriomegaly type (Hollier classification) and management were recorded. Patients with incomplete data sets were excluded from the study.

## Results

A total of 2,592 conventional angiograms, 5 CT angiograms and 2,677 MRA scans were performed during the study period. A diagnosis of arteriomegaly was made in 69 patients (1.5%). The diagnostic modality was conventional angiography in 52 patients and MRA in 57 patients. Sixty-seven of the patients were men and two were women. Data from two male patients’ notes were incomplete and therefore not included. The mean age of the study cohort was 74 years (range: 60–89 years). Risk factors found in these patients are summarised in Table 1.

**Table 1:** Risk factors for patients with arteriomegaly(n=67)

Smoking	49 (73%)
Myocardial disease	38 (57%)
Hypertension	29 (43%)
Diabetes mellitus	18 (27%)
Cerebrovascular accident	7 (10%)
Family history	7 (10%)

The mode of presentation of these patients varied: 49 patients (75%) were admitted with critical limb ischaemia and 18 patients (27%) were referred to the vascular clinic with symptoms of intermittent claudication. The diagnosis at initial presentation is summarised in Table 2.

**Table 2:** Diagnosis at initial presentation

Diagnosis	Number
Aortic aneurysm	20
Common lilac stenosis	2
External Iliac occlusion	2
Common femoral artery occlusion	4
Superficial femoral artery occlusion	20
Superficial femoral artery aneurysm	2
Popliteal artery aneurysm	9
Multi-level stenosis	7

**Figure 1:**
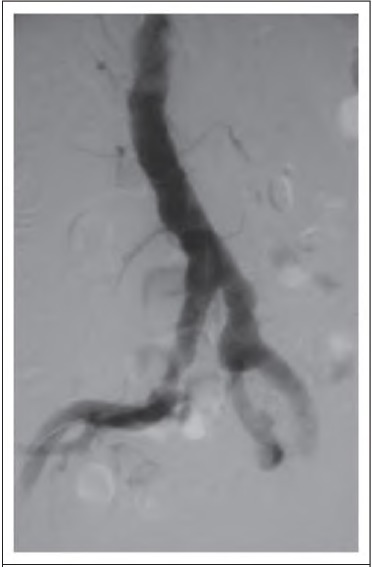
Angiogram showing aortolllac segment of a patient with Holllertype I arteriomegaly

The Hollier classification was used to determine the type of arteriomegaly at presentation and to monitor the progression of the disease. At presentation, 51 patients had arteriomegaly but no aneurysmal disease, 22 patients were classified as Hollier type I, 5 as type II and 9 as type III (Figs 1^–^4). Management at initial presentation was conservative in all 51 patients with arteriomegaly but no aneurysmal disease, in 2 patients with type I disease and 1 patient with type II disease. Six patients needed percutaneous transluminal angioplasty (PTA) on the same admission. Twenty-seven patients needed surgical intervention.

**Figure 2:**
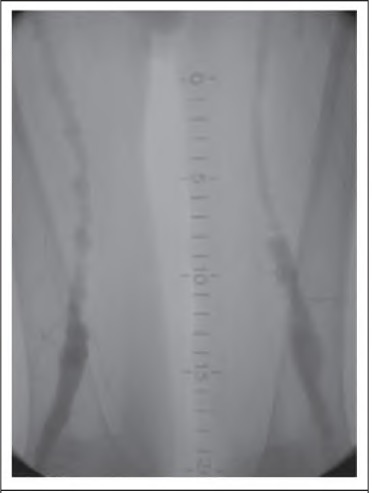
Angiogram showing femoropopllteal segment of a patient with Holllertype II arteriomegaly

**Figure 3:**
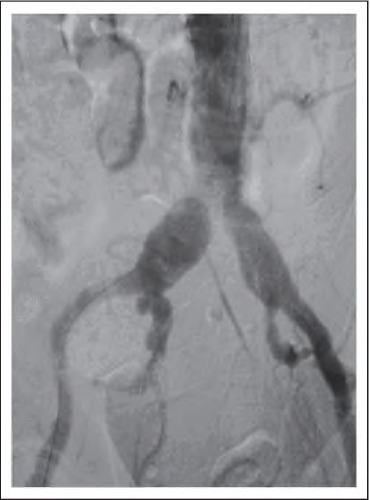
Angiogram showing aortoiiiac segment of a patient with Hoiiiertype III arteriomegaly

**Figure 4:**
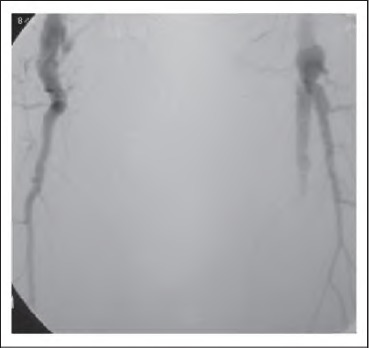
Angiogram showing femoropopllteal segment of a patient with Holllertype III arteriomegaly

All patients were followed up in the vascular outpatients clinic. The follow-up duration was for a median of 76 months (range: 6–146 months). During that period, the progression of the disease became evident (Table 3). After a median follow up of 76 months, 54 patients had progressed to type I, 2 to type II and 18 to type III. Thirteen remained without aneurysmal disease.

**Table 3:** Progression of patients with arteriomegaly (Hollier’s classification)

Type	Initial classification	Classification at follow up
Nil	31	13
Type 1	22	34
Type 11	5	2
Type III	9	18

As the disease progressed, patients required further intervention. Thirty-one patients underwent PTA and twentyeight had bypass surgery. The surgical procedures that type I, type II and type III arteriomegalics underwent are shown in Tables 4–6. During the follow-up period, our cohort of 67 patients required a total of 102 procedures for complicated disease. Thirty-eight patients required two or more surgeries for complications. This was due to thrombotic events in 9 patients, graft infection in 1 patient and concomitant occlusive disease in 10 patients. Five patients in the group treated conservatively initially developed complications necessitating surgical intervention. This was due to vascular occlusion in all cases. There were no cases of ecstatic vessel rupture.

**Table 4:** Surgical procedures for type I arteriomegaly

Procedure	Number
Abdominal aortic aneurysm repair	7
Femoro-femoral crossover bypass	5
Aorto-bllllac graft	2
Femoropopllteal graft	12
Femoral aneurysmectomy	2
Endarterectomy	5
Femorotlblal bypass	4
Major amputation	2
Minor amputation	2
Angioplasty	16

**Table 5:** Surgical procedures for type II arteriomegaly

Procedure	Number
Popliteal aneurysm repair	2
Graft revision	1
Angioplasty	7

**Table 6:** Surgical procedures for type III arteriomegaly

Procedure	Number
Femoropopllteal bypass	6
Abdominal aortic aneurysm repair	6
Major amputation	3
Embolectomy	2
Exploration groin	1
Primary aneurysm repair	8
Angioplasty	5

**Table 7:** Procedures for arteriomegaly without aneurysmal disease

Procedure	Number
Embolectomy	2
Angioplasty	2

After a median follow-up duration of 76 months, there had been 8 deaths (12%). Of these, two patients died during the postoperative period from sepsis and the remaining six died during follow up from non-vascular causes, all during the first ten months from diagnosis.

The limb salvage rate was 92%; five lower limbs were amputated after operative surgery failed to restore limb perfusion.

## Discussion

The first description of an elongated and tortuous splenic artery was in 1571 by Julius Caesar Aranzi of Vienna (‘arteriae lienis, ductum obliquum ac flexosum’). In 1945 René Leriche first reported arteriomegaly as elongated and enlarged arteries.^1^ Later, Lea Thomas described arteriomegaly, or arteria magna, as non-aneurysmal enlargement of arteries associated with the tortuosity and markedly slow arterial flow seen on arteriogram.^3^

The reported incidence ranges between 1.6% and 11% in the population undergoing imaging of the lower limbs.^2^, ^4^

The lower incidence of our study may be a result of our strict defining terminology as described by the Hollier classification. The risk factors for arteriomegaly remain similar to that of abdominal aortic aneurysm formation. It is associated with connective tissue disorders, metabolic disorders and fibromuscular dysplasias.^5^ In addition, it is closely related to multiple and contiguous aneurysms,^6^ especially in the popliteal region, which are reported to occur in about 50–60% of arteriomegalics.^2^, ^7^ Two small studies described an incidence of 57–60% aneurysms in arteriomegalic patients.^3^, ^7^ Lawrence *et al* suggested a familial association with aneurysmal disease.^8^ They examined 14 patients with arteriomegaly and found a 56% familial incidence of abdominal aortic aneurysm. Indeed, some authors have suggested that screening family members of patients affected by arteriomegaly and/or peripheral arterial aneurysms would be beneficial.^4^

Clinically, arteriomegalic patients can suffer chronic claudication or acute limb ischaemia secondary to an increased risk of thromboembolism and aneurysmal degeneration.^4^ Pathologically, the disruption of the inner elastic layer results in elongation and tortuosity.^9^ Specimens obtained in several studies revealed specific morphologic changes distinct from those of the atherosclerotic change. In these, there was no significant damage to myocytes and only a mild increase in microfibril number and degeneration of the elastic tissue with fragmentation of the elastic layer.^5^, ^10^

This view has been challenged, however. Yamamoto *et al* found no microscopic differences in the aneurysm walls of arteriomegalic compared to non-arteriomegalic vessels.^11^ It has been suggested that in addition to atherosclerotic stimuli, other unknown systemic or constitutional factors are involved in the formation of aneurysms associated with arteriomegaly, in contrast to those not associated with arteriomegaly.^11^ This is supported by findings that peripheral arteries, usually resistant to atherosclerotic disease, such as the brachial and distal external carotid arteries are dilated in aneurysmal disease relative to controls.^12^ These results support the notion of a generalised dilating diathesis in aortic aneurysmal disease that may be independent of atherosclerosis.

Criteria to define ecstatic vessels and aneurysmal vessels have been based on the size of the arteries on angiography. This has its limitations. Radiography magnifies and distorts the diseased arteries and is therefore not a true representation of the artery. Furthermore, the flow of contrast in arteriomegalic vessels is slow and hence assessment is hindered. Some studies have shown that flow can be so slow as to make assessment of distal run-off difficult.^4^, ^7^, ^13^, ^14^

Arteriomegaly appears to be a disease of elderly men. The average age in our cohort was 74 years. Studies have suggested that arteriomegaly may begin at an earlier age than abdominal aortic aneurysm disease or that the increased frequency of complications brings them to medical attention earlier.^2^, ^15^ The study by Hollier *et al* comprised a series of 91 men.^2^ There were no female disease sufferers. The mean age in the series was 67.5 years. In our series of 67 patients, we had two female patients. It seems to therefore be an almost exclusively male disease.

During the follow-up period, the progressive nature of this arteriopathy becomes evident. The majority of patients who had no aneurysmal disease at presentation developed multiple aneurysms at different levels of the aorta-iliac-femoral tree. During follow up, our cohort required a total of 102 procedures for complicated disease processes. The propensity to progress makes the decision making of when to intervene difficult. Hollier *et al* are in favour of offering extensive arterial reconstruction to avoid postoperative complications due to the fact that the disease is left untreated.^2^ In their series of 91 male patients, a total of 19 patients underwent 21 amputations; 17 patients required no surgery but 74 patients required a total of 169 operations. Over half the patients (51%) required more than one operative procedure. Reoperation was required in 20% of type II and 49% of type III patients. No reoperation was needed in type I patients.

In addition to the three types of arteriomegaly described by Hollier, we suggest a fourth type is introduced, describing the presence of arteriomegaly without aneurysmal de generation. In our study, 58% of patients with type IV arteriomegaly progressed to more advanced disease requiring surgical management. These patients also demonstrate a progressive disease process that deserves special management and follow up.

Our study supports the evidence that arteriomegaly is associated with a high rate of thrombotic complications.^2^, ^16^ We encountered thrombotic events as the presenting complication in 58.8% of patients with arteriomegaly («=26). Hollier *et al* do not see a role for thrombectomy or local resection of a thrombosed segment of arterial vessel.^2^ They believe that this may be associated with a high rate of reocclusion from the proximal artery containing laminated thrombus. They adopt an approach of more extensive revascularisation to prevent this complication.

Our method is more conservative. Our approach to localised disease is with angioplasty, thrombectomy or local resection where feasible. Bypass surgery is reserved for those patients unsuitable for local procedures. AH patients require close and frequent follow up regardless of intervention. As arteriomegaly is a multivessel disease, it is to be expected that patients will need multiple procedures when the aneurysms are large enough or symptomatic. Accordingly, most of our patients have had multiple operations and/or PTA. At present, our mortality rate is comparable to the study by Hollier *et al.^2^* The limb salvage in our series is greater.

There are several drawbacks to our observational study. Firstly, the modality of arteriography for diagnosis of arteriomegaly varied. Conventional angiography has limitations as it can only evaluate luminal size and inaccuracies may therefore have occurred. However, as MRA has only superseded conventional angiography in more recent years, this is unavoidable. Secondly, as we are a district general hospital, we currently do not have access to emergency or ‘out-ofhours’ endovascular procedures although this is presently in development. As such, our management of patients may change in favour of endovascular procedures for occlusive disease when such services become available. Management decisions may hence differ in hospitals where endovascular services are available.

## Conclusions

Arteriomegaly is a progressive disease affecting the arterial tree. It is identified in 1.5% of patients undergoing lower limb angiography. The disease is associated with diffuse aneurysmal changes. The natural history of the disease is not well understood. The results of our more conservative approach to the treatment of the disease in our institution are similar to those of studies that adopt a more aggressive approach. Multiple procedures are often required to prevent complications and regular follow-up visits for disease progression are therefore important.

Local resection and bypass surgery followed by regular follow-up appointments and early diagnosis and intervention is an acceptable option to more extensive radical surgery. We support the notion that the management of aortofemoral aneurysmal disease together with arteriomegaly is potentially different from patients without arteriomegaly. We propose an additional ‘type IV’ category to Hollier’s classification that describes diffuse arteriomegaly without aneurysmal changes. These patients also demonstrate progressive disease. The existence of arteriomegaly is a significant sign in the diagnosis, management and follow up of patients.
